# 137. Comparison of genotypic resistance pattern between *Enterococcus faecium* and *Enterococcus faecalis* to better understand the occurrence of mutations.

**DOI:** 10.1093/ofid/ofac492.215

**Published:** 2022-12-15

**Authors:** Sowmya Padakanti, Vismay Badhiwala, Munok Hwang, Hosoon Choi, Piyali Chatterjee, Sorabh Dhar, Keith S Kaye, Chetan Jinadatha

**Affiliations:** Baylor Scott and White Hospital, Temple, Texas; Baylor Scott & White Hospital, Temple, Texas; Central Texas Veterans Health Care System, Temple, Texas; Central Texas Veterans Health Care System, Temple, Texas; Central Texas Veterans Health Care System, Temple, Texas; Wayne State University, Detroit, Michigan; Rutgers - Robert Wood Johnson Medical School, New Brunswick, New Jersey; Central Texas Veterans Health Care System, Temple, Texas

## Abstract

**Background:**

*Enterococcus* is one of the major causes of nosocomial infections many with vancomycin resistance. About 50,000 infections are reported per year due to vancomycin-resistant *Enterococci*. Understanding the genotypic mutations can help recognize the potential burden for antibiotic resistance that exists in a population. In this study, using whole genome sequencing (WGS) we aim to analyze the prevalence of genetic resistance markers for commonly used antibiotics for *Enterococcal* treatment.

**Methods:**

Whole genome sequencing was performed using the NextSeq (Illumina Inc., CA) on 60 isolates of *E. faecium* and 29 isolates of *E. faecalis*. The isolates were obtained from two different Detroit area hospitals from 2017-2019. The data from WGS was analyzed using EPISEQ CS^TM^ (BIOMÉRIEUX, Marcy l ‘Etoile, France) bioinformatic database to obtain specific resistance genes which would correspond to commonly used antibiotics for treatment of *Enterococcal* infections.

**Results:**

Among the 89 *Enterococcus faecium* and *Enterococcus faecalis* investigated, we identified a total of 33 unique resistance genes across 18 classes of antibiotics (Table 1). We detected genetic mutations *efmA, pbp5, tet(L), liar, liaS* unique to *E. faecium* and only *emeA* unique to *E. faecalis*. *E. faecium* and *E. faecalis* share 14 similar resistance genes. *E. faecium* and *E. faecalis* had 12 and 7 unique resistance genes, respectively but are of same drug classes. The most common antibiotic classes include aminoglycosides, tetracyclines, quinolones, beta-lactams, glycopeptides, macrolides, and pyrimidine analogs.

**Table 1:**
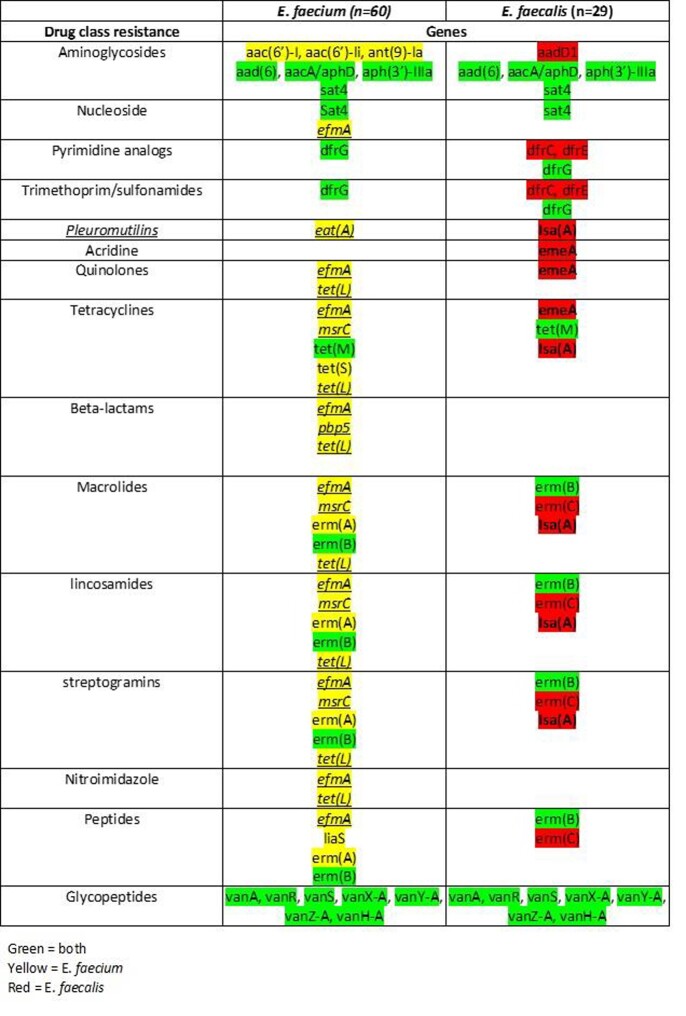
Enterococcus faecium and E. faecalis genetic mutations and drug class resistance

**Conclusion:**

*Enterococcus faecium* had a greater number of resistance genes with glycopeptide, aminoglycoside, and beta lactam resistance when compared to *Enterococcus faecalis*. Whereas *Enterococcus faecalis* had more resistance genes associated with macrolides, tetracyclines, pyrimidine analogs, and Bactrim. In general, both appear to have many genes that encode for resistance to major classes of antibiotics which are used for treatment of *Enterococcal* infections. Further comparison with phenotypic susceptibility testing data would help better understand the practical implications of our resistance gene testing.

**Disclosures:**

**Piyali Chatterjee, PhD**, AHRQ Grant # 1R03HS027667-01: Grant/Research Support|AHRQ Grant # 1R03HS027667-01: Central Texas Veterans Health Care System **Keith S. Kaye, MD, MPH**, Allecra: Advisor/Consultant|GlaxoSmithKline plc.: Receiving symposia honoraria|GlaxoSmithKline plc.: GlaxoSmithKline plc.-sponsored study 212502|Merck: Advisor/Consultant|qpex: Advisor/Consultant|Shionogi: Grant/Research Support|Spero: Advisor/Consultant **Chetan Jinadatha, MD, MPH**, AHRQ R01 Grant-5R01HS025598: Grant/Research Support|EOS Surfaces: Copper Coupons and materials for testing.

